# Telomere components as potential therapeutic targets for treating microbial pathogen infections

**DOI:** 10.3389/fonc.2012.00156

**Published:** 2012-11-01

**Authors:** Bibo Li

**Affiliations:** Center for Gene Regulation in Health and Disease, Department of Biological, Geological, and Environmental Sciences, Cleveland State UniversityCleveland, OH, USA

**Keywords:** telomere, virulence, microbial pathogen, antigenic variation, infectious diseases

## Abstract

In a number of microbial pathogens that undergoes antigenic variation to evade the host’s immune attack, genes encoding surface antigens are located at subtelomeric loci, and recent studies have revealed that telomere components play important roles in regulation of surface antigen expression in several of these pathogens, indicating that telomeres play critical roles in microbial pathogen virulence regulation. Importantly, although telomere protein components and their functions are largely conserved from protozoa to mammals, telomere protein homologs in microbial pathogens and humans have low sequence homology. Therefore, pathogen telomere components are potential drug targets for therapeutic approaches because first, most telomere proteins are essential for pathogens’ survival, and second, disruption of pathogens’ antigenic variation mechanism would facilitate host’s immune system to clear the infection.

## INTRODUCTION

Telomeres are nucleoprotein complexes located at the ends of linear chromosomes. In most eukaryotic cells, telomere DNA consists of simple repetitive TG-rich sequences and is maintained by telomerase, a ribonucleoprotein that contains both a catalytic protein subunit and an RNA component providing the template for *de novo* telomere DNA synthesis (Greider and Blackburn, 1987).

A number of proteins have been identified that specifically associate with the telomere DNA. In mammalian cells, the core telomere protein complex termed “Shelterin” (de Lange, 2005) contains two duplex TTAGGG repeat binding factors, TRF1 and TRF2 (Chong et al., 1995; Bilaud et al., 1997; Broccoli et al., 1997), a single-stranded telomere DNA binding protein, POT1 (Baumann and Cech, 2001), and RAP1 (Li et al., 2000), TIN2 (Kim et al., 1999), and TPP1 (Houghtaling et al., 2004; Liu et al., 2004; Ye et al., 2004) that interact with TRFs or POT1. In addition, a trimeric CST complex containing CTC1, STN1, and TEN1 has also been identified to bind the single-stranded telomere DNA (Miyake et al., 2009; Wan et al., 2009). Fission yeast has a very similar telomere protein complex (Miyoshi et al., 2008), and many telomere protein homologs have been identified in budding yeast, too (Lewis and Wuttke, 2012). Recent studies have led to the identification of TRF (Li et al., 2005) and RAP1 (Yang et al., 2009) homologs in *Trypanosoma brucei*, a protozoan parasite belongs to the kinetoplastids group, suggesting that the telomere complex is largely conserved from protozoan to mammalian cells. Telomeres, together with their associated protein components, form a specialized structure so that the natural chromosome ends are properly protected (Stewart et al., 2011), while maintenance of a stable length of telomere DNA provides adequate docking sites for telomere binding proteins. Therefore, telomeres are essential for genome stability and sustained cell proliferation.

Although telomeres are predominantly maintained by telomerase in most eukaryotes, DNA homologous recombination can also serve as an important means for telomere maintenance (McEachern and Haber, 2006; Nabetani and Ishikawa, 2011). In addition, subtelomeric DNA recombination appears to be a major factor for genome plasticity, which may help to diversify the sequences of subtelomeric genes (Corcoran et al., 1988; Pologe and Ravetch, 1988; De Bruin et al., 1994; Louis, 1995). For several microbial pathogens whose virulence genes are located next to telomeres, this can also be an important pathogenesis mechanism to enhance their virulence (see below).

A telomere position effect (TPE) phenomenon has been observed in a number of organisms, where the expression of genes located at subtelomeres is suppressed by the nearby telomere chromatin structure (Gottschling et al., 1990; Baur et al., 2001; Koering et al., 2002; Park et al., 2002; Pedram et al., 2006). TPE is well studied in *S. cerevisiae*, where ScRap1 binds the duplex telomere DNA (Longtine et al., 1989). Both ScRap1 and yKu (a heterodimer complex that binds DNA ends in a sequence-independent manner; Riha et al., 2006) can recruit the Sir4 silencer to the telomere, and ScRap1 can also recruit Sir3 (Moretti et al., 1994; Martin et al., 1999; Moretti and Shore, 2001; Luo et al., 2002). Together, Sir3 and Sir4 recruit Sir2 (Moazed et al., 1997; Strahl-Bolsinger et al., 1997; Buchberger et al., 2008; Martino et al., 2009), which is an NAD^+^-dependent histone deacetylase (Tanny et al., 1999; Landry et al., 2000) and can remove the acetyl group from histone H3 at K9 and K14 residues and from histone H4K16 (Imai et al., 2000). Sir2 activity and the interaction between Sir3/4 and histone tails are necessary for establishing and propagating of the heterochromatic structure from telomere to chromosome internal regions (Hoppe et al., 2002). Similarly, TPE in human cells appears to be mediated by the heterochromatic chromatin structure, as treatment with Trichostatin A, an inhibitor of class I and II histone deacetylases, led to decreased TPE (Koering et al., 2002).

## ANTIGENIC VARIATION AND PHENOTYPIC SWITCH IN MICROBIAL PATHOGENS

Many microbial pathogens that infect mammals have adopted antigenic variation to avoid eradication by the host immune system so that they can maintain persistent infections and enhance the chances of being transmitted to new hosts. Antigenic variation is the phenomenon that a pathogen changes its surface antigen presented to the host immune system regularly and much more frequently than spontaneous gene mutation. The term of antigenic variation usually encompasses both phase variation and true antigenic variation. In phase variation (such as phenotypic switching), the expression of an individual antigen switches between “on” or “off” states. Multiple genes from the same gene family can be expressed at the same time, and each gene’s expression state is relatively independent to that of other genes in the same family. Phenotypic switching can contribute to the virulence of the pathogen because expressing different types or various number of surface molecules may enhance or weaken adhesion of the pathogen to the host. In true antigenic variation, a certain antigen switches among different forms. The antigen is usually expressed in a mutually exclusive manner – a single gene from a multi-copy gene family is expressed at any time. In general, both antigenic variation and phenotypic switching can occur through two general types of mechanisms: genetic and epigenetic (Deitsch et al., 2009). A genetic event involves changes in DNA sequences of an antigen encoding gene or its regulatory elements so that either its expression level or its gene product is changed. An epigenetic event only affects a gene expression level but does not change its DNA sequences. However, recent studies suggest that epigenetic changes such as chromatin remodeling may also influence genetic events such as DNA recombination (Benetti et al., 2007; Bisht et al., 2008). Common mechanisms of antigenic variation have evolved in different pathogens, including bacteria, fungi, and parasites, possibly due to similar selection pressure exerted from the mammalian immune responses. However, in this chapter, we will focus on those mechanisms that are influenced or likely to be affected by the telomere structure.

## TPE PARTICIPATES IN THE REGULATION OF *EPA* EXPRESSION IN *C. glabrata*

*Candida glabrata* is part of the normal human mucosal flora and usually commensal, but it can cause opportunistic mucosal and bloodstream infections in immunocompromised individuals. During infection, binding of the pathogen to host cell proteins or microbial competitors would help to reduce the chance of clearance by the host. Therefore, the adherence of *C. glabrata* to host cells has been proposed to play an important role in its virulence (Kaur et al., 2005).

**FIGURE 1:**
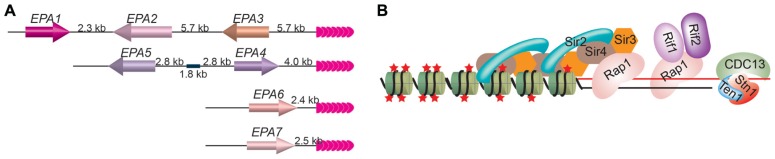
**(A)** *EPA1*–*7* are located at subtelomeric loci in *C. glabrata.* The positions of seven *EPA* genes at their respective chromosome end loci are shown. *EPA1* is furthest away from the telomere and is the only one that is expressed normally, while *EPA 2–7* are usually silenced by TPE. Pink arrowheads, telomere repeats. **(B)** The telomere protein complex in budding yeast. Rap1 is the duplex telomere DNA binding factor, while Cdc13/Stn1/Ten1 binds to single-stranded telomere DNA. Rap1 recruits Sir3 and Sir4, which in turn recruits Sir2. Sir3 and Sir4 can also interact with histones directly. Sir2’s deacetylase activity maintains the hypoacetylated state of histones. Rap1 also recruits Rif1 and Rif2. Red stars, histone acetylation groups; green cylinders, nucleosomes.

When cultured human epithelial cells are used, 95% of *in vitro C. glabrata* adherence depends on an adhesin molecule that binds the host *N*-acetyl lactosamine-containing glycoconjugates (Castano et al., 2005) and is encoded by the *EPA1* gene (Kapteyn et al., 1999), which belongs to the *EPA* gene family. So far, a total of 23 putative *EPA* genes and pseudogenes have been identified in *C.glabrata* strain BG2 based on their sequence similarity (Kaur et al., 2005). Seven *EPA* genes encode full-length GPI-anchored proteins, among which Epa1 is a lectin (Cormack et al., 1999), Epa6 and Epa7 are confirmed to be adhesins (Castano et al., 2005), and Epa2 and Epa3 are predicted to be cell wall proteins (De Las Penas et al., 2003). All seven *EPA* genes located at subtelomeric regions (**Figure 1**; De Las Penas et al., 2003; Castano et al., 2005; Iraqui et al., 2005).

Normally, only *EPA1* gene is active, while *EPA2–7* genes are silenced by TPE, which depends on telomere protein Rap1. Deletion of the C-terminal 28 amino acids of Rap1 led to derepression of *EPA4–7* and sometimes also *EPA2* and *EPA3 *(De Las Penas et al., 2003). Silencing of subtelomeric *EPA* genes also depends on Sir proteins (De Las Penas et al., 2003; Castano et al., 2005). Deletion of *SIR3* led to hyper expression of *EPA1* and derepression of *EPA2–7*, although the derepression of *EPA2, EPA3, *and *EPA4/5* is mild. Deletion of *SIR4* also led to derepression of *EPA6*. In the case of deletion of *SIR3*, expression of *EPA6* and *EPA7* appears to contribute to the hyper-adherent phenotype, indicating that TPE can be directly involved in regulation of pathogen virulence (Castano et al., 2005). Interestingly, Epa6 expression is associated with the ability of *C. glabrata* cells to form biofilm on plastic surface (Iraqui et al., 2005). Biofilm formed by microbial pathogens can increase infection probability and is of great clinical importance because microorganisms adopting this life form is more tolerant or resistant to host defense machinery and anti-microbial agents than free cells.

This TPE regulated adhesin expression is well exploited by *C. glabrata* to adapt to the host environment. *C. glabrata* is an nicotinic acid (NA or vitamin niacin) auxotroph, as it lost all the *BNA* genes involved in the NA synthesis except *BNA5 *(Domergue et al., 2005). When growing in urine, where NA is limited, the activity of Sir2, an NAD^+^-dependent histone deacetylase, decreases correspondingly since NA is the precursor of NAD^+^. As a consequence, TPE level decreases, and *EPA1*, *6*, and *7 *genes are highly expressed (Domergue et al., 2005). This effect can be reverted by adding NA or a related compound nicotinamide (NAM). Most importantly, when using an established murine model of urinary track infection, transurethrally inoculated *C. glabrata* has an elevated colonization frequency in bladder and kidney, which is dependent on *EPA1, 6*, and *7* gene expression, and mice fed with high-NA diet are no longer susceptible to high rate of colonization of *C. glabrata *(Domergue et al., 2005). Therefore, in *C. glabrata*, TPE plays an important role in regulation of virulence gene expression.

## Sir2-MEDIATED TPE PLAYS AN ESSENTIAL ROLE IN MANOALLELIC EXPRESSION OF *var* GENES IN *P. falciparum*

*Plasmodium falciparum* is a protozoan parasite in the Apicomplexa phylum that causes the most severe form of malaria, which is a debilitating and sometimes fatal disease mostly found in tropical and subtropical regions of the world. During *P. falciparum *infection in a human host, the parasite invades first hepatocytes then erythrocytes. One major reason why it is very difficult to eliminate these parasites once an infection is established is that *P. falciparum* undergoes antigenic variation at the erythrocyte stage (Dzikowski and Deitsch, 2009). At this stage, *P. falciparum *cells produce erythrocyte membrane protein 1 (PfEMP1), which is encoded by *var* genes and is transported to the infected erythrocyte membrane (Baruch et al., 1995; Smith et al., 1995; Su et al., 1995). Expression of PfEMP1 on the infected cell surface allows the infected erythrocyte to adhere to the endothelium of the post-capillary venules and avoid circulation through the spleen, where the infected cells will be destroyed (Baruch, 1999). Therefore, expression of PfEMP1 on host cell surface is critical for prolonged parasite infection. However, PfEMP1 is also susceptible to host antibody recognition and subsequent immune attack. As an important virulence mechanism, *P. falciparum* regularly switches the expressed PfEMP1, therefore effectively evading the host immune attack (Roberts et al., 1992).

**FIGURE 2:**
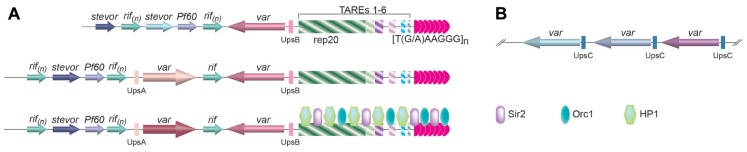
**(A)** The organization of subtelomere elements in *P. falciparum.* Immediately internal to the telomere tract are six telomere-associated repeat elements (TAREs 1*–*6), with the largest one, *rep20*, located furthest away from the telomere repeats. One or two *var* genes are usually found immediately upstream of *rep20*, followed by the *rifin*, *stevor*, and *Pf60* gene families. Depending on the upstream flanking sequences, three classes of *var* genes have been identified. The ones with associated UpsB and UpsA are located at subtelomeric regions and transcribed in opposite directions as drawn, while the ones with associated UpsC are located as gene arrays inchromosome-internal loci **(B)**. Little is known about the telomere proteins in *Plasmodium*, except that Sir2 and Orc1 is located at the telomere vicinity (Mancio-Silva et al., 2008), and HP1 is associated with subtelomeric TAREs (Perez-Toledo et al., 2009), which are shown in the bottom diagram in**(A)**.

There are ~60 *var* genes in the *P. falciparum* genome (Gardner et al., 2002). However, only one *var* gene is expressed at any moment (Roberts et al., 1992). Based on its upstream regulatory elements, *var* genes can be classified into three groups (**Figure2**; Kraemer and Smith, 2003; Lavstsen et al., 2003). Those with UpsA and transcribed toward the telomere and those with UpsB and transcribed away from the telomere are located at subtelomeric loci (**Figure 2A**), while the ones with UpsC are located at chromosome internal loci (**Figure 2B**; Voss et al., 2000; Gardner et al., 2002; Kraemer and Smith, 2003; Lavstsen et al., 2003). Monoallelic expression of *var* gene is regulated at multiple levels, and telomeres appear to play an important role (Dzikowski and Deitsch, 2009).

Telomere position effect in *P. falciparum* spreads ~55kb along the chromosome from telomeres and was first observed by targeting a reporter gene to the rep20 repeats located at the subtelomeric regions (**Figure 2A**; Duraisingh et al., 2005). Rep20 is the most telomere-distal telomere associated repetitive element (TARE) and is usually adjacent to the subtelomeric *var* gene promoter. TPE in *P. falciparum* depends on Sir2 (Duraisingh et al., 2005; Freitas-Junior et al., 2005; Tonkin et al., 2009), which is both a histone deacetylase and an ADP-ribosyltransferase (Merrick and Duraisingh, 2007; Chakrabarty et al., 2008) and is localized at the telomeres, where histones H4 acetylation is absent (Freitas-Junior et al., 2005). By examining subnuclear localization of a number of genetic markers along chromosome 2 by FISH, it is also inferred that chromatin structure is more condensed for telomere-proximal regions than telomere-distal ones (Freitas-Junior et al., 2005). The direct evidence of involving TPE in *var* gene regulation came from the observation that deletion of PfSir2 led to a significant increase in transcription of a subset of *var* genes, particularly the *var* genes with UpsA and at the subtelomere regions (Duraisingh et al., 2005).

## RAP1-MEDIATED SILENCING IS ESSENTIAL FOR MONOALLELIC EXPRESSION OF *VSG* IN *T. brucei*

The kinetoplastids are a group of flagellated protozoa. Three members of kinetoplastids are of great clinical importance because they cause human diseases: *Trypanosoma brucei* causes human African trypanosomiasis or sleeping sickness, *Trypanosoma cruzi *causes South America trypanosomiasis or Chagas disease, and several *Leishmania* species cause leishmaniasis. Of these three trypanosomatids (the kinetoplastid organisms that only have a single flagellum), only *T. brucei* undergoes antigenic variation, which is an important mechanism of its pathogenesis and one of its most interesting biological aspects (Barry and McCulloch, 2001).

*Trypanosoma brucei* is transmitted between mammalian hosts by an insect vector, tsetse (*Glossina *spp.). While inside the mid-gut of a tsetse fly, *T. brucei* cells are in the non-virulent proliferative stage, procyclic form (PF), and several procyclic acidic repetitive proteins (PARPs, or procyclins) are expressed on its surface. After *T. brucei* cells migrate into the salivary gland of the tsetse fly, they differentiate into the metacyclic form, stop proliferating, and acquire virulence. When a tsetse fly takes a blood meal, *T. brucei* cells can be injected into a mammalian host. They stay in the bloodstream or extracellular spaces in the host and quickly differentiate into bloodstream form (BF). The slender BF is proliferative, while the stumpy form is quiescent and non-proliferative. The metacyclic form and BF cells express variant surface glycoproteins (VSGs) as their major surface glycoprotein (Mehlert et al., 1998). When a tsetse fly bites the infected mammalian host, stumpy bloodstream form *T. brucei* cells taken into the midgut of a tsetse can quickly differentiate into the PF, ending the life cycle (Matthews, 2005).

*Trypanosoma brucei *is exposed to the host’s immune system and is vulnerable to both the innate (inflammations, complements, etc.) and adaptive immune responses (antibody, killer T cells, etc.). However, *T. brucei* has evolved a sophisticated antigenic variation mechanism and regularly switches its surface VSG coat, thus effectively evading the host’s immune response (Barry and McCulloch, 2001).

Antigenic variation in *T. brucei* has two essential aspects: switch to express a different *VSG* gene (*VSG* switching) and monoallelic expression of *VSG*. Although there are >1,500 *VSG* genes and pseudogenes in the *T. brucei* genome (Berriman et al., 2005), only one type of VSG is expressed at any time. After a new *VSG* gene is turned on, it is essential to turn off the previously active *VSG* so that the old surface antigen is no longer presented to the host immune system. In addition, expressing only one *VSG* gene at a time would allow the *VSG* gene pool to be used for a maximum period of time, enabling a persistent infection. Therefore, both *VSG* switching and monoallelic expression of *VSG* are critical for antigenic variation and have been the focus of intensive research for several decades.

**FIGURE 3:**
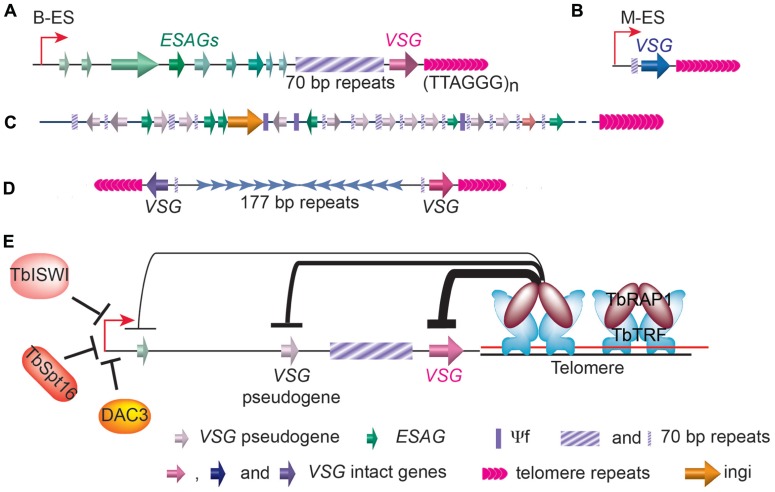
**Distribution of *VSG* genes in *T. brucei* genome**. **(A)** In a bloodstream form *VSG* expression site (B-ES), the *VSG* gene is the last one in the large polycistronic transcription unit and is located within 2kb of the telomere repeats. A stretch of 70bp repeats with various length is located upstream of the *VSG* gene followed by a number of ES associated genes (*ESAGs*). **(B)** The metacyclic *VSG* expression site (M-ES) is a monocistronic transcription unit also located at subtelomeric region. **(C)** Most *VSG* genes and pseudogenes (and some *ESAG* genes) are found in gene arrays located at subtelomeric regions on megabase chromosomes. Short stretches of 70bp repeats are found upstream of each gene. **(D)** On minichromosomes, single *VSG* genes and upstream 70bp repeats are also found at subtelomeric regions. **(E)** The telomere protein, TbRAP1, has been shown to play an important role in silencing subtelomeric *VSG* genes. TbTRF and TbRAP1 are two known *T. brucei* telomere proteins. TbRAP1-mediated silencing is stronger (thick line) at telomere-proximal *VSG* locus and weaker (thin line) at telomere-distal ES promoter region. Several factors important for ES promoter silencing are also shown.

There are 11 pairs of megabase chromosomes (0.9–5.7Mb), several intermediate chromosomes (300–900kb), and ~100 copies of minichromosomes (50–100kb) in *T. brucei* genome (Melville et al., 2000; Alsford et al., 2001; Berriman et al., 2005). The majority of *VSG* genes are found in long tandem arrays of repeated genes at subtelomeric locations on megabase chromosomes (**Figure 3C**). Approximately 200 copies of *VSG* genes are found immediately upstream of telomeres of the minichromosomes, which carry besides the *VSG* genes, only repetitive sequences, including 177bp repeats in the chromosome internal region and telomere repeats at the chromosome ends (**Figure 3D**; Alsford et al., 2001). The *VSG* genes in subtelomeric gene arrays and on minichromosome are often referred to as basic *VSG* copies because they are transcriptional silent. The rest of* VSGs* are found in *VSG* expression sites. In BF *T. brucei* cells, *VSGs* are expressed exclusively from bloodstream form *VSG* expression sites (B-ESs) which are RNA polymerase I (RNAP I)-transcribed, polycistronic transcription units located at subtelomere loci (**Figure 3A**; de Lange and Borst, 1982; Gunzl et al., 2003). *VSG* is the last gene in any B-ES and is usually within 1.5kb from the telomere repeats, while the promoter is often 40–60kb upstream of the *VSG *(Hertz-Fowler et al., 2008). In contrast, at the metacyclic stage, *VSGs* are expressed from metacyclic *VSG* expression sites (M-ESs), which are monocistronic transcription units located at the subtelomeric regions (**Figure 3B**), with the promoter located only ~5kb from the telomere (Lenardo et al., 1984; Cornelissen et al., 1985). Although the M-ESs have much simpler organizations than the B-ESs, much less is understood about metacyclic than bloodstream *VSG* expression regulation. *T. brucei* has multiple B-ESs (e.g., Lister 427 has 15 different B-ESs), usually carrying different *VSGs*, but all B-ESs have very similar genomic organization with ~90% sequence identity (Hertz-Fowler et al., 2008). Earlier studies focused on B-ES promoters also showed that they are almost always identical (Zomerdijk et al., 1990, 1991; Pham et al., 1996). Therefore, how *T. brucei* manages to fully express only one B-ES and *VSG* had been a great puzzle for more than a couple of decades.

A number of studies in the last decade have shown that *VSG* expression is regulated at multiple levels. First, transcription elongation from B-ES promoter appears to be regulated. Silent B-ES promoters are actually mildly active (Vanhamme et al., 2000), but transcription elongation is quickly attenuated after a few kilobases, effectively stopping transcription long before the *VSG* genes. Second, chromatin structure of the active B-ES is very different from the silent B-ESs. The active B-ES has very few nucleosomes while silent ESs are packed with nucleosomes (Figueiredo and Cross, 2010; Stanne and Rudenko, 2010). Chromatin remodeling also plays an important role in regulating the B-ES expression: Deletion of the histone H3K79 methyltransferase TbDot1b led to a 10-fold increase in transcription throughout the silent B-ESs (Janzen et al., 2006). Additional chromatin remodelers have been shown to affect B-ES promoter but not downstream *VSG* expression (**Figure 3E**): Depletion of a Swi/Snf homolog, TbISWI, led to an elevated transcription from the silent B-ES promoters (Hughes et al., 2007; Stanne et al., 2011); DAC3, a histone deacetylase homologs, is required for B-ES promoter silencing (Wang et al., 2010); and depletion of TbSpt16, a subunit of the FACT chromatin remodeling complex, also led to an ~20-fold increase in silent B-ES promoter transcription (Denninger et al., 2010). Third, ever since the discovery that *VSGs* are exclusively expressed from subtelomeric regions (de Lange and Borst, 1982), it has been proposed that telomeres may play an important role in *VSG* expression regulation (Dreesen et al., 2007). This hypothesis was supported by the fact that TPE has been observed in *T. brucei *(Horn and Cross, 1997a; Glover and Horn, 2006).

Although the earlier studies provided promising evidence for TPE, direct evidence linking TPE and *VSG* silencing was lacking for a long time. In addition, although the *T. brucei* Sir2 homolog plays an essential role in TPE at reporter marked telomeres without native B-ESs, its deletion does not affect *VSG* silencing at all (Alsford et al., 2007). Furthermore, Glover et al. (2007) was able to target an I-Sce I digestion site together with a *neo* reporter gene downstream of the *VSG* gene and immediately upstream of the telomere in a telomerase null background. Induction of ectopic I-SceI expression led to immediate cleavage and loss of the marked telomere. Within 9h, degradation of the reporter gene and the subtelomeric *VSG* gene was also observed. Although a mild derepression of the reporter gene was observed shortly before it was degraded, the *VSG* gene was not derepressed at all (Glover et al., 2007). These observations raised a great deal of doubts whether telomeres are indeed necessary for proper *VSG* silencing.

It was difficult to examine the roles of the telomere in antigenic variation directly without identifying any telomere specific proteins. Earlier attempts to identify telomere DNA binding factors in *T. brucei* using biochemical approaches led to the identification of a couple of telomere DNA binding activities without identification of the responsible proteins (Eid and Sollner-Webb, 1995, 1997).

The first *T. brucei* Shelterin homolog, TbTRF, was identified using an *in silico* approach (Li et al., 2005), and a yeast 2-hybrid screen using TbTRF as bait led to the identification of *T.brucei* RAP1 (Yang et al., 2009), another integral component of the *T.brucei* telomere complex (**Figure 3E**). When TbRAP1 was depleted by RNAi (Shi et al., 2000), a derepression of silent B-ES-linked *VSGs *can be detected (Yang et al., 2009). Using quantitative RT-PCR analysis, it was shown that all B-ES-linked silent* VSGs* had an elevated expression level upon depletion of TbRAP1, although the level of derepression varies among different *VSGs*, ranging from 8- to 56-fold. Subsequently, it was confirmed by IF that multiple VSGs are expressed simultaneously in individual cells on cell surface (Yang et al., 2009). Importantly, the TbRAP1-mediated silencing is position dependent. First, only subtelomeric B-ES-linked *VSGs* were affected. Genes located in chromosome internal regions including RNAP I transcribed rDNA and RNAP II transcribed telomerase protein gene, a ribosomal protein gene, and a glycolytic protein gene were not affected. Second, within an individual B-ES, the telomere-adjacent *VSG* gene is derepressed at the highest level, a *VSG* pseudogene located 7–20kb away from the telomere is derepressed at an intermediate level, and a reporter gene targeted immediately downstream of the B-ES promoter located 40–60kb away from the telomere is derepressed at the lowest level (**Figure 3E**). It is therefore convinced that the TbRAP1-mediated silencing originates from the telomere, demonstrating for the first time that the telomere structure indeed plays an essential role in *VSG* expression regulation (Yang et al., 2009).

However, the involvement of telomere in *VSG* expression regulation does not necessarily exclude other mechanisms mentioned above. In fact, TbRAP1-mediated silencing appears to block the elongation of the basal level transcription from the silent B-ES promoters, because in TbRAP1 deficient cells, derepressed *VSGs* are expressed at a level that is still ~100 fold lower than when the same *VSG* is in a fully active B-ES (Yang et al., 2009). Therefore, the observed quick attenuation of transcription elongation along silent B-ESs may well be the combined effect of a basal level transcription initiated from silent B-ES promoters and a TbRAP1-mediated TPE. The fact that derepressed *VSGs *are not expressed at its fullest potential also suggests that B-ES promoters are regulated by additional factors other than TPE. This is consistent with the observations that a number of chromatin remodeling factors are involved in B-ES promoter regulation as mentioned above.

Recent studies have made great contributions to our understanding of how *VSG* expression is silenced. However, how is allelic-exclusive expression of *VSG* achieved is not fully understood. It has been proposed that sufficient amount of RNAP I machinery, which is responsible for high level *VSG* transcription, may be accessible to only one B-ES, which would effectively ensure its monoallelic expression (Horn and McCulloch, 2010). In an IF analysis, Navarro and Gull (2001) found that in BF *T. brucei* cells, transcriptionally active RNAP I forms a small nuclear focus in addition to the large focus inside the nucleolus, where it transcribes rRNA. In addition, only the active B-ES but not the silent ones is co-localized with RNAP I in this ES body (ESB), which only exists in BF but not PF cells (Navarro and Gull, 2001). It is therefore hypothesized that ESB, enriched with RNAP I, can only accommodate one B-ES, which would effectively limit the number of active B-ES to one. In support of this view, when two different B-ESs were tagged with selective markers immediately downstream of their respective promoters and forced to be active simultaneously, the two B-ESs appear to switch back and forth rapidly and locate next to each other in the nucleus, presumably competing for available RNAP I at ESB (Chaves et al., 1999).

## TELOMERE LENGTH AFFECTS *VSG* SWITCHING FREQUENCY AND MECHANISM IN *T. brucei*

**FIGURE 4:**
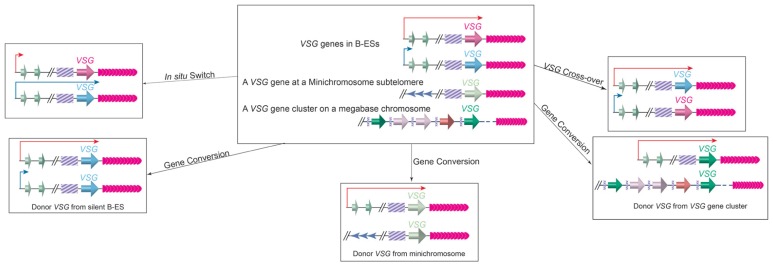
***VSG* switching can occur through *in situ* switch, gene conversion, or crossover**. Top Middle, before switching, an active B-ES (long red arrow), a silent B-ES (short blue arrow), a *VSG* gene at a minichromosome subtelomere, and an array of *VSG* genes and pseudogenes on a megabase chromosome are shown. *In situ* switch (top left) results from turning on (long blue arrow) of the silent B-ES and turning off (short red arrow) of theactive B-ES simultaneously without any DNA rearrangements. In gene conversion, a silent *VSG* gene is duplicated into the active B-ES, and the originally active *VSG* gene is lost. The *VSG* donor can come from a silent B-ES (bottom left), a minichromosome subtelomere (bottom middle), or a *VSG* gene array (bottom-right). In *VSG* cross-over (top right), the active *VSG* and a silent *VSG* (most often from a silent B-ES) exchange their loci reciprocally, resulting in a new *VSG* gene in the active B-ES without losing any genetic information. The cross-over site is often found within the 70bp repeats upstream of the *VSG* genes, although it can locate more upstream, and all B-ESs have high sequence homology.

*VSG* switching can occur through several different pathways (**Figure 4**; Barry and McCulloch, 2001). In the so-called *in situ* switch, a silent B-ES promoter is turned fully active while the originally active B-ES promoter is turned off without any DNA rearrangements. There are 15 B-ESs carrying distinctive *VSGs* in the *T. brucei* Lister 427 cells, providing a small number of possible *in situ* switch opportunities (Hertz-Fowler et al., 2008). However, *in situ VSG* switching is usually a rare event, and *VSG* switching involving DNA recombination events are much more prevalent (Robinson et al., 1999). The large *VSG *gene pool, therefore, provides essentially endless possibilities for *VSG* switching.

In gene conversion events, a silent *VSG* is copied into the active B-ES while the originally active *VSG* is lost. In this event, the donor can be any functional *VSG* gene in the genome. There is almost always a stretch of 70bp repeats upstream of a *VSG* gene, in which homologous recombination can initiate as DNA double-strand breaks (Boothroyd et al., 2009). In rare occasions, several *VSG* donors have been identified in a single *VSG* switching event, where each donor contributes only a fragment of the gene, generating a new mosaic *VSG* gene product (Marcello and Barry, 2007). Such mechanism has been proposed to be useful in late stage of persistent infection. More often, a silent B-ES is used as a donor possibly because long stretch of 70bp repeats (2 to >14kb) and telomere repeats (3–20kb) flank the *VSG* gene in any B-ES, and efficient homologous recombination can initiate from these sites. In fact, all B-ESs have very similar genome organization and are ~90% identical in sequences, so gene conversion event can initiate at places upstream of 70bp repeats and often a whole silent B-ES can be copied to replace the active B-ES (Pays et al., 1983b; Hertz-Fowler et al., 2008). Therefore, the terms of *VSG* gene conversion and ES gene conversion are used to differentiate different types of gene conversion events (Kim and Cross, 2010). In addition to gene conversion, reciprocal crossover event can occur in a *VSG* switching (Rudenko et al., 1996). In this case, the crossover usually occurs at the 70bp repeats, and the silent and active *VSG*s (often together with their respective downstream telomeres) simply trade places without deletion of large fragments of genetic information. It is worth to note that in a crossover switching, the originally silent *VSG* often comes from a silent B-ES, but it can also be from a minichromosome subtelomere. Finally, more complicated switching events involving loss of the active B-ES or *VSG* associated with an *in situ* switch have also been observed (Kim and Cross, 2011).

It has been shown that homologous DNA recombination is important for *VSG* switching in *T. brucei *(McCulloch and Barry, 1999). In homologous recombination, searching for DNA sequence homology and subsequent strand-invasion is a key step, at which RAD51 polymerizes around ssDNA to assemble a nucleoprotein helical filament and, with the help of ATP, extends the DNA structure and carries out the strand exchange process (Holloman, 2011). When ssDNA is coated with RPA (a single-strand-specific DNA binding protein), it will not be accessible by RAD51 without the help of a mediator, such as BRCA2 (Holloman, 2011). In *T.brucei*, six RAD51 related proteins have been identified: RAD51, DMC1, RAD51-3, RAD51-4, RAD51-5, and Rad51-6 (Proudfoot and McCulloch, 2005). Among these, deletion of TbRAD51 and TbRAD51-3 led to a decrease in *VSG* switching rate while deletion of TbRAD51-5 did not have any effect, and deletion of TbBRCA2 also led to a similar decreased *VSG* switching rate (Hartley and McCulloch, 2008). In addition, TbTOPO3α and TbRMI1, whose homologs in mammalian cells form a so-called RTR complex with The RecQ helicase BLM and suppress aberrant and inappropriate homologous recombination, were recently shown to be involved in regulation of *VSG* switching (Kim and Cross, 2010, 2011).

Apparently, homologous recombination is a major pathway for *VSG* switching. However, exactly how *VSG* switching is regulated is less clear. Several recent studies now indicate that the telomere structure can influence *VSG* switching greatly.

It has been shown that the active *VSG*-marked telomere is less stable than the silent telomeres (Bernards et al., 1983; Pays et al., 1983a; van der Ploeg et al., 1984; Myler et al., 1988; Horn and Cross, 1997b). Rapidly shortened active telomere arises frequently, which is quite similar to the TRD observed in yeast cells carrying abnormally long telomeres (Li and Lustig, 1996). Presumably the active transcription of the telomere is a major cause for the brittle active telomere (Rudenko and Van der Ploeg, 1989). With the presence of telomerase, shortened telomeres are elongated quickly (Horn et al., 2000). With frequent truncation and elongation, telomere length at the active chromosome end is often much more heterogeneous than those at silent telomeres (Bernards et al., 1983). However, in the absence of telomerase, the truncated active telomere remains short, allowing the isolation of clones baring extremely short active telomere in a relatively short culturing period (Dreesen and Cross, 2006). Interestingly, when such telomerase negative clones were obtained that carry extremely short active telomere, these clones tend to switch to express a new *VSG *(Dreesen and Cross, 2006). This observation led to the hypothesis that shorter telomeres may cause higher *VSG* switching rate (Dreesen et al., 2007). It is speculated that all active telomeres are prone to large telomere fragment deletions due to its active transcription state, but shorter telomeres are more likely to have a deletion landed in the subtelomeric region and to cause damage in the active *VSG* gene, which will force the parasite to go through *VSG* switching. Introducing a break at the I-SceI site targeted immediately upstream of the active *VSG* gene led to a 250-fold increase in *VSG* switching frequency, confirming part of this theory that damage to the active *VSG* gene will force the parasite to switch (Boothroyd et al., 2009).

Importantly, a recent study showed that cells carrying short active telomeres (~1.5kb) has an ~6.3-fold higher *VSG* switching frequency than cells carrying long telomeres (>10kb; Hovel-Miner et al., 2012). In addition, cells with short active telomere also have more gene conversion and much fewer telomere crossover events as *VSG* switching mechanism (Hovel-Miner et al., 2012). Therefore, telomere length indeed affects subtelomeric *VSG* switching. At least two Shelterin homologs have been identified in *T. brucei *(Li et al., 2005; Yang et al., 2009), which enabled further investigation of the telomere structure in *VSG* switching regulation. It is speculated that disruption of the heterochromatic telomere structure, especially in the case of depletion of TbRAP1 (Yang et al., 2009), may also lead to higher *VSG* switching rate, similar to what was observed in *S. pombe* (Bisht et al., 2008).

## DOES TELOMERE AFFECT SWITCHING OF SUBTELOMERE-LOCATED SURFACE ANTIGEN IN *P. carinii* AND *B. burgdorferi*?

*Pneumocystis carinii* is a fungus that solely dwells in the lung tissue of mammals. Normally, *P. carinii *infection does not cause any symptom, but in immunocompromised individuals it can cause pneumonia. The complete life cycle of *P. carinii *is still not very well defined, mainly because of the lack of a continuous cultivation system. However, it is obvious that *P. carinii *can survive in the lower respiratory tract where strong and effective defense systems normally work to eliminate invaders, and the reason for persistent and effective *P. carinii *infection is that it undergoesantigenic variation at a high frequency (Cushion and Stringer, 2010).

**FIGURE 5:**
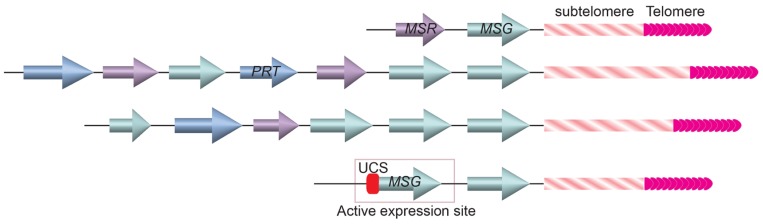
**Gene arrays at the ends of three *Pneumocystis carinii *chromosomes**. *MSG* genes (cyan colored arrows) are located closest to the telomere and subtelomeric repetitive sequences. A single copy UCS is found in the active *MSG* expression site immediately upstream of the *MSG* gene.

The Major Surface Glycoprotein (MSG) is one of the major surface molecules of *P. carinii *that is involved in antigenic variation (Stringer, 2005). MSG is encoded by the *MSG* gene family. So far 73 *MSG* genes have been identified, all are located at the subtelomeric loci (**Figure 5**; Keely and Stringer, 2009). There are 17 chromosomes in *P. carinii *(Hong et al., 1990), indicating that on average at least two *MSG* genes are at each telomere, which is often the case in cloned terminal fragments from various chromosomes (Wada and Nakamura, 1996; Keely et al., 2005). Similar to the situation in *T. brucei*, only one *MSG* gene is transcribed at any time. Transcribed *MSG* messengers always contained an upstream conserved sequence (UCS; Wada et al., 1995; Edman et al., 1996; Wada and Nakamura, 1996; Sunkin and Stringer, 1997), which has only one copy in the *P. carinii *genome (Wada et al., 1995; Edman et al., 1996), suggesting that *MSG* is transcribed from a specific expression site marked with the unique UCS element. In addition, translation initiation codon on an *MSG* mRNA is located in the sequence transcribed from the UCS (Wada et al., 1995; Edman et al., 1996). Therefore, transcribing MSG from UCS-containing expression site is essential for proper MSG translation. Furthermore, the UCS encoded peptide contains a signal sequence that targets the pre-MSG protein into the endoplasmic reticulum, where it can be cleaved and glycosylated, then deposited on the cell surface (Sunkin et al., 1998). Hence the UCS peptide is also essential for MSG function, although it is not present on MSG found on the cell surface because it is likely removed in the endoplasmic reticulum.

If *P. carinii *contains only one UCS-containing *MSG* expression site, how does it achieve antigenic variation? Computational analysis of *MSG* gene sequences suggested that these genes commonly undergo recombination (Wada and Nakamura, 1996; Keely et al., 2005; Keely and Stringer, 2009), which is not unlike the *VSG* switching in *T. brucei*. Similar to *VSG*, *MSG* is also the last transcribed gene on the chromosome (Wada and Nakamura, 1996; Keely et al., 2005). The proximity of *MSG* genes to telomeres suggests that the *MSG* switching events might also be regulated by the telomere structure, although this has not be investigated at all.

**FIGURE 6:**
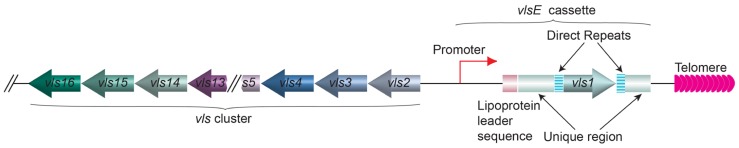
**The organization of *vlsE* and the array of silent *vls* genes on lp28 linear plasmid of *B. burgdorferi***. The *vls1* gene (moss colored arrow) is expressed from the *vlsE* expression site next to the telomere (pink arrows). The direct repeats (barred boxes) and the unique regions (green boxes) flanking the *vls1* gene and the lipoprotein leader sequence (rouge box) upstream of *vls1* are marked. The silent array includes *vls2–16* genes (various colored arrows) going to the opposite direction from *vls1* are located at the internal region of the linear plasmid.

In a different microbial pathogen *Borrelia burgdorferi*, the spirochete that causes the Lyme disease, the gene encoding variant surface antigen is found at a subtelomere region on a linear plasmid (Zhang et al., 1997). *B. burgdorferi* also undergoes antigenic variation, and the lipoprotein VlsE is the variant surface protein (Schwan et al., 1991; Zhang et al., 1997; Zhang and Norris, 1998b; Steere et al., 2004; Norris, 2006). VlsE is encoded by the *vls* gene family located on the linear plasmid lp28-1 (**Figure 6**). Immediately next to the telomere is the active *vlsE* expression site. More upstream is the silent *vls* gene cluster (Zhang et al., 1997). Bacteria lost the lp28-1 exhibit an intermediate infectivity phenotype where it is hard to establish a persistent infection in the mouse model (Bankhead and Chaconas, 2007). Deletion of *vlsE* and silent *vls* cassettes also led to reduced persistent infection, indicating that antigenic variation through *vls* switching is an important virulence mechanism in *B. burgdorferi *(Zhang et al., 1997; Purser and Norris, 2000; Labandeira-Rey and Skare, 2001; Bankhead and Chaconas, 2007). The *vlsE* and the silent *vls* genes are highly homologous at the sequence level, and most of the sequence differences within the cassette regions are concentrated in six variable regions, VR1–VR6 (Zhang and Norris, 1998a). Segmental gene conversion between the silent cassettes and the *vlsE* cassette region occurs as early as 4days after infection in mice, and appears to continue throughout the course of infection (Zhang and Norris, 1998b). Because these recombination events appear to involve random segments of any silent cassette and occur continuously during infection, an almost unlimited number of *VlsE* amino acid sequence permutations are theoretically possible (Zhang and Norris, 1998a). Apparently, *vls* switching is not so unlike the *VSG* switching in *T. brucei* or *MSG* switching in *P. carinii*. However, nothing is known about the telomere structure at the ends of lp28 or any protein(s) associated with it. Therefore, it is unclear whether the nearby telomere structure might exhibit any influence to *vls* switching.

## TELOMERE COMPONENTS AS POTENTIAL TARGETS OF ANTI-PATHOGEN AGENTS

In this review, I have provided a detailed treatise of the telomere region, the adjoining genes and sites, and the regulatory elements and proteins in several microbial pathogens that undergo antigenic variation. As discussed, telomere forms a specialized heterochromatic structure that can influence the expression of genes located nearby. It appears that several microbial pathogens have conveniently taken advantage of this TPE to regulate expression of surface antigen-encoding gene families at subtelomeric regions. Further studies of the telomere structure and telomere-specific proteins in these microbial pathogens should provide more insight about the allelic exclusion expression of surface antigen genes. In addition, the subtelomeric region in many eukaryotic cells appears to be a DNA recombination hot spot, presumably contributing to gene diversity. This could be one of the reasons why many gene families encoding virulence factors are located at subtelomeric loci in microbial pathogens. One cannot help to speculate that the intrinsic plastic nature of the subtelomeres might facilitate antigenic variation. On the other hand, unchecked homologous recombination could cause hazardous genome instability, and the telomere structure with telomere-specific proteins is hypothesized to suppress subtelomeric recombination to maintain a relatively stable genome organization.

The essential functions of telomeres in maintaining genome integrity are conserved for all eukaryotic cells, and homologs of many telomere-specific proteins have been identified from protozoa to mammals. However, telomere homologs from mammals and those from the above-mentioned microbial pathogens have very low sequence homology. As the telomere components play important functions in regulation of virulence in several microbial pathogens, they are attractive drug targets for treatment of diseases caused by these pathogens. For example, TbRAP1 and hRAP1 has very limited sequence homology. It should be feasible to identify or develop agents that specifically target TbRAP1 but not hRAP1. These agents are expected to act as a double-edged sword. First, TbRAP1 is essential for trypanosome cell growth and dysfunctional TbRAP1 leads to cell growth arrest. Second, TbRAP1 is essential for complete subtelomeric *VSG* silencing, and lack of TbRAP1 leads to expression of multiple VSG proteins on trypanosome cell surface, which will facilitate the host immune system to eliminate the parasite efficiently.

Because most microbial pathogens grow much faster than their mammalian host, they are also more susceptible to agents that disrupt the telomere structure or cause telomere length attrition, which is more detrimental to fast growing cells. Because *T. brucei* has a 3′ single-stranded G-rich overhang at the end of the telomeres, compounds that target the G-quadruplex such as Imetelstat would be a good choice to inhibit *T. brucei* growth preferentially. Imetelstat is a lipid-conjugated oligonucleotide (previously known as GRN163), with excellent tissue penetration, bioavailability, and efficacy that has been used to against a variety of cancers (Dikmen et al., 2005; Herbert et al., 2005; Gellert et al., 2006). Other small molecule inhibitors that interact with the human telomeric DNA are available (Sun et al., 1997), but they may not inhibit parasites other than *T. brucei *due to telomere sequence dissimilarity. Another potential target would be telomerase that synthesize the telomere DNA in most eukaryotic cells. Although knockout telomerase is not expected to cause immediate deleterious effects in parasites such as *T. brucei* that carry long telomeres, changing the telomere sequence by incorporation of mutations into the telomerase RNA template may lead to more acute cell growth arrest due to disrupted binding of the mutant telomere DNA by normal telomere binding proteins, such as TRF.

We have just begun to understand the functions of telomeres in antigenic variation. New telomere components are continuously investigated for their potential roles in this important mechanism of pathogenesis. As we gain more knowledge, we expect to identify more suitable telomere components as good anti-pathogen targets.

## Conflict of Interest Statement

The author declares that the research was conducted in the absence of any commercial or financial relationships that could be construed as a potential conflict of interest.

## References

[B1] Alsford S. Kawahara T. Isamah C. Horn D. (2007). A sirtuin in the African trypanosome is involved in both DNA repair and telomeric gene silencing but is not required for antigenic variation. *Mol. Microbiol.* 63 724–736 17214740 10.1111/j.1365-2958.2006.05553.x

[B2] Alsford S. Wickstead B. Ersfeld K. Gull K. (2001). Diversity and dynamics of the minichromosomal karyotype in *Trypanosoma brucei*. *Mol. Biochem. Parasitol.* 113 79–88 11254956 10.1016/s0166-6851(00)00388-1

[B3] Bankhead T. Chaconas G. (2007). The role of VlsE antigenic variation in the Lyme disease spirochete: persistence through a mechanism that differs from other pathogens. *Mol. Microbiol.* 65 1547–1558 17714442 10.1111/j.1365-2958.2007.05895.x

[B4] Barry J. D. McCulloch R. (2001). Antigenic variation in trypanosomes: enhanced phenotypic variation in a eukaryotic parasite. *Adv. Parasitol.* 49 1–70 11461029 10.1016/s0065-308x(01)49037-3

[B5] Baruch D. I. Pasloske B. L. Singh H. B. Bi X. Ma X. C. Feldman M. (1995). Cloning the *P. falciparum* gene encoding PfEMP1, a malarial variant antigen and adherence receptor on the surface of parasitized human erythrocytes. *Cell* 82 77–87 7541722 10.1016/0092-8674(95)90054-3

[B6] Baruch D. I. (1999). Adhesive receptors on malaria-parasitized red cells. *Baillieres Best Pract. Res. Clin. Haematol.* 12 747–761 10895262 10.1053/beha.1999.0051

[B7] Baumann P. Cech T. R. (2001). Pot1, the putative telomere end-binding protein in fission yeast and humans. *Science* 292 1171–1175 11349150 10.1126/science.1060036

[B8] Baur J. A. Zou Y. Shay J. W. Wright W. E. (2001). Telomere position effect in human cells. *Science* 292 2075–2077 11408657 10.1126/science.1062329

[B9] Benetti R. Gonzalo S. Jaco I. Schotta G. Klatt P. Jenuwein T. (2007). Suv4-20h deficiency results in telomere elongation and derepression of telomere recombination. *J. Cell Biol.* 178 925–936 17846168 10.1083/jcb.200703081 PMC2064618

[B10] Bernards A. Michels P. A. M. Lincke C. R. Borst P. (1983). Growth of chromosome ends in multiplying trypanosomes. *Nature* 303 592–597 6304531 10.1038/303592a0

[B11] Berriman M. Ghedin E. Hertz-Fowler C. Blandin G. Renauld H. Bartholomeu D. C. (2005). The genome of the African trypanosome *Trypanosoma brucei*. *Science* 309 416–422 16020726 10.1126/science.1112642

[B12] Bilaud T. Brun C. Ancelin K. Koering C. E. Laroche T. Gilson E. (1997). Telomeric localization of TRF2, a novel human telobox protein. *Nat. Genet.* 17 236–239 9326951 10.1038/ng1097-236

[B13] Bisht K. K. Arora S. Ahmed S. Singh J. (2008). Role of heterochromatin in suppressing subtelomeric recombination in fission yeast. *Yeast* 25 537–548 18615848 10.1002/yea.1603

[B14] Boothroyd C. E. Dreesen O. Leonova T. Ly K. I. Figueiredo L. M. Cross G. A. M. (2009). A yeast-endonuclease-generated DNA break induces antigenic switching in *Trypanosoma brucei*. *Nature* 459 278–281 19369939 10.1038/nature07982 PMC2688456

[B15] Broccoli D. Smogorzewska A. Chong L de Lange T. (1997). Human telomeres contain two distinct Myb-related proteins, TRF1 and TRF2. *Nat. Genet.* 17 231–235 9326950 10.1038/ng1097-231

[B16] Buchberger J. R. Onishi M. Li G. Seebacher J. Rudner A. D. Gygi S. P. (2008). Sir3-nucleosome interactions in spreading of silent chromatin in *Saccharomyces cerevisiae*. *Mol. Cell. Biol.* 28 6903–6918 18794362 10.1128/MCB.01210-08 PMC2573294

[B17] Castano I. Pan S. J. Zupancic M. Hennequin C. Dujon B. Cormack B. P. (2005). Telomere length control and transcriptional regulation of subtelomeric adhesins in *Candida glabrata*. *Mol. Microbiol.* 55 1246–1258 15686568 10.1111/j.1365-2958.2004.04465.x

[B18] Chakrabarty S. P. Saikumari Y. K. Bopanna M. P. Balaram H. (2008). Biochemical characterization of *Plasmodium falciparum* Sir2, a NAD^+^-dependent deacetylase. *Mol. Biochem. Parasitol.* 158 139–151 18221799 10.1016/j.molbiopara.2007.12.003

[B19] Chaves I. Rudenko G. Dirks-Mulder A. Cross M. Borst P. (1999). Control of variant surface glycoprotein gene-expression sites in * Trypanosoma brucei*. *EMBO J.* 18 4846–4855 10469662 10.1093/emboj/18.17.4846 PMC1171556

[B20] Chong L. van Steensel B. Broccoli D. Erdjument-Bromage H. Hanish J. Tempst P. (1995). A human telomeric protein. *Science* 270 1663–1667 7502076 10.1126/science.270.5242.1663

[B21] Corcoran L. M. Thompson J. K. Walliker D. Kemp D. J. (1988). Homologous recombination within subtelomeric repeat sequences generates chromosome size polymorphisms in *P. falciparum*. *Cell* 53 807–813 3286016 10.1016/0092-8674(88)90097-9

[B22] Cormack B. P. Ghori N. Falkow S. (1999). An adhesin of the yeast pathogen *Candida glabrata* mediating adherence to human epithelial cells. *Science* 285 578–582 10417386 10.1126/science.285.5427.578

[B23] Cornelissen A. W. Bakkeren G. A. Barry J. D. Michels P. A. Borst P. (1985). Characteristics of trypanosome variant antigen genes active in the tsetse fly. *Nucleic Acids Res.* 13 4661–4676 4022771 10.1093/nar/13.13.4661 PMC321818

[B24] Cushion M. T. Stringer J. R. (2010). Stealth and opportunism: alternative lifestyles of species in the fungal genus *Pneumocystis*. *Annu. Rev. Microbiol.* 64 431–452 20528694 10.1146/annurev.micro.112408.134335

[B25] De Bruin D. Lanzer M. Ravetch J. V. (1994). The polymorphic subtelomeric regions of *Plasmodium falciparum* chromosomes contain arrays of repetitive sequence elements. *Proc. Natl. Acad. Sci. U.S.A.* 91 619–623 8290573 10.1073/pnas.91.2.619 PMC43000

[B26] de Lange T. (2005). Shelterin: the protein complex that shapes and safeguards human telomeres. *Genes Dev.* 19 2100–2110 16166375 10.1101/gad.1346005

[B27] de Lange T. Borst P. (1982). Genomic environment of the expression-linked extra copies of genes for surface antigens of *Trypanosoma brucei* resembles the end of a chromosome. *Nature* 299 451–453 7121582 10.1038/299451a0

[B28] De Las Penas A. Pan S. J. Castano I. Alder J. Cregg R. Cormack B. P. (2003). Virulence-related surface glycoproteins in the yeast pathogen *Candida glabrata* are encoded in subtelomeric clusters and subject to RAP1- and SIR-dependent transcriptional silencing. *Genes Dev.* 17 2245–2258 12952896 10.1101/gad.1121003 PMC196462

[B29] Deitsch K. W. Lukehart S. A. Stringer J. R. (2009). Common strategies for antigenic variation by bacterial, fungal and protozoan pathogens. *Nat. Rev. Microbiol.* 7 493–503 19503065 10.1038/nrmicro2145 PMC3676878

[B30] Denninger V. Fullbrook A. Bessat M. Ersfeld K. Rudenko G. (2010). The FACT subunit TbSpt16 is involved in cell cycle specific control of VSG expression sites in *Trypanosoma brucei*. *Mol. Microbiol.* 78 459–474 20879999 10.1111/j.1365-2958.2010.07350.x PMC3034197

[B31] Dikmen Z. G. Gellert G. C. Jackson S. Gryaznov S. Tressler R. Dogan P. (2005). *In vivo* inhibition of lung cancer by GRN163L: a novel human telomerase inhibitor. *Cancer Res*. 65 7866–7873 16140956 10.1158/0008-5472.CAN-05-1215

[B32] Domergue R. Castano I. De Las Penas A. Zupancic M. Lockatell V. Hebel J. R. (2005). Nicotinic acid limitation regulates silencing of *Candida adhesins* during UTI. *Science* 308 866–870 15774723 10.1126/science.1108640

[B33] Dreesen O Cross G. A. M. (2006). Consequences of telomere shortening at an active VSG expression site in telomerase-deficient *Trypanosoma brucei*. *Eukaryot. Cell* 5 2114–2119 17071826 10.1128/EC.00059-06 PMC1694812

[B34] Dreesen O. Li B Cross G. A. M. (2007). Telomere structure and function in trypanosomes: a proposal. *Nat. Rev. Microbiol.* 5 70–75 17160000 10.1038/nrmicro1577

[B35] Duraisingh M. T. Voss T. S. Marty A. J. Duffy M. F. Good R. T. Thompson J. K. (2005). Heterochromatin silencing and locus repositioning linked to regulation of virulence genes in *Plasmodium falciparum*. *Cell* 121 13–24 15820675 10.1016/j.cell.2005.01.036

[B36] Dzikowski R. Deitsch K. W. (2009). Genetics of antigenic variation in *Plasmodium falciparum*. *Curr. Genet.* 55 103–110 19242694 10.1007/s00294-009-0233-2 PMC3640992

[B37] Edman J. C. Hatton T. W. Nam M. Turner R. Mei Q. Angus C. W. (1996). A single expression site with a conserved leader sequence regulates variation of expression of the *Pneumocystis carinii* family of major surface glycoprotein genes. *DNA Cell Biol.* 15 989–999 8945640 10.1089/dna.1996.15.989

[B38] Eid J. E. Sollner-Webb B. (1995). ST-1, a 39-kilodalton protein in *Trypanosoma brucei*, exhibits a dual affinity for the duplex form of the 29-base-pair subtelomeric repeat and its C-rich strand. *Mol. Cell. Biol.* 15 389–397 7799947 10.1128/mcb.15.1.389 PMC231977

[B39] Eid J. E. Sollner-Webb B. (1997). ST-2, a telomere and subtelomere duplex and G-strand binding protein activity in *Trypanosoma brucei*. *J. Biol. Chem.* 272 14927–14936 9169464 10.1074/jbc.272.23.14927

[B40] Figueiredo L. M Cross G. A. M. (2010). Nucleosomes are depleted at the VSG expression site transcribed by RNA polymerase I in African trypanosomes. *Eukaryot. Cell* 9 148–154 19915072 10.1128/EC.00282-09 PMC2805297

[B41] Freitas-Junior L. H. Hernandez-Rivas R. Ralph S. A. Montiel-Condado D. Ruvalcaba-Salazar O. K. Rojas-Meza A. P. (2005). Telomeric heterochromatin propagation and histone acetylation control mutually exclusive expression of antigenic variation genes in malaria parasites. *Cell* 121 25–36 15820676 10.1016/j.cell.2005.01.037

[B42] Gardner M. J. Hall N. Fung E. White O. Berriman M. Hyman R. W. (2002). Genome sequence of the human malaria parasite *Plasmodium falciparum*. *Nature* 419 498–511 12368864 10.1038/nature01097 PMC3836256

[B43] Gellert G. C. Dikmen Z. G. Wright W. E. Gryaznov S. Shay J. W. (2006). Effects of a novel telomerase inhibitor, GRN163L, in human breast cancer. *Breast Cancer Res. Treat.* 96 73–81 16319992 10.1007/s10549-005-9043-5

[B44] Glover L. Alsford S. Beattie C. Horn D. (2007). Deletion of a trypanosome telomere leads to loss of silencing and progressive loss of terminal DNA in the absence of cell cycle arrest. *Nucleic Acids Res.* 35 872–880 17251198 10.1093/nar/gkl1100 PMC1807955

[B45] Glover L. Horn D. (2006). Repression of polymerase I-mediated gene expression at *Trypanosoma brucei* telomeres. *EMBO Rep.* 7 93–99 16311518 10.1038/sj.embor.7400575 PMC1369228

[B46] Gottschling D. E. Aparicio O. M. Billington B. L. Zakian V. A. (1990). Position effect at *S. cerevisiae* telomeres: reversible repression of pol II transcription. * Cell* 63 751–762 2225075 10.1016/0092-8674(90)90141-z

[B47] Greider C. W. Blackburn E. H. (1987). The telomere terminal transferase of *Tetrahymena* is a ribonucleoprotein enzyme with two kinds of primer specificity. *Cell* 51 887–898 3319189 10.1016/0092-8674(87)90576-9

[B48] Gunzl A. Bruderer T. Laufer G. Schimanski B. Tu L. C. Chung H. M. (2003). RNA polymerase I transcribes procyclin genes and variant surface glycoprotein gene expression sites in *Trypanosoma brucei*. *Eukaryot. Cell* 2 542–551 12796299 10.1128/EC.2.3.542-551.2003 PMC161450

[B49] Hartley C. L. McCulloch R. (2008). *Trypanosoma brucei* BRCA2 acts in antigenic variation and has undergone a recent expansion in BRC repeat number that is important during homologous recombination. *Mol. Microbiol.* 68 1237–1251 18430140 10.1111/j.1365-2958.2008.06230.x PMC2408642

[B50] Herbert B. S. Gellert G. C. Hochreiter A. Pongracz K. Wright W. E. Zielinska D. (2005). Lipid modification of GRN163, an N3′→P5′ thio-phosphoramidate oligonucleotide, enhances the potency of telomerase inhibition. *Oncogene* 24 5262–5268 15940257 10.1038/sj.onc.1208760

[B51] Hertz-Fowler C. Figueiredo L. M. Quail M. A. Becker M. Jackson A. Bason N. (2008). Telomeric expression sites are highly conserved in *Trypanosoma brucei*. *PLoS ONE* 3 e3527 10.1371/journal.pone.0003527 PMC2567434 18953401

[B52] Holloman W. K. (2011). Unraveling the mechanism of BRCA2 in homologous recombination. *Nat. Struct. Mol. Biol.* 18 748–754 21731065 10.1038/nsmb.2096 PMC3647347

[B53] Hong S. T. Steele P. E. Cushion M. T. Walzer P. D. Stringer S. L. Stringer J. R. (1990). *Pneumocystis carinii* karyotypes. *J. Clin. Microbiol.* 28 1785–1795 1975595 10.1128/jcm.28.8.1785-1795.1990 PMC268048

[B54] Hoppe G. J. Tanny J. C. Rudner A. D. Gerber S. A. Danaie S. Gygi S. P. (2002). Steps in assembly of silent chromatin in yeast: Sir3-independent binding of a Sir2/Sir4 complex to silencers and role for Sir2-dependent deacetylation. *Mol. Cell. Biol.* 22 4167–4180 12024030 10.1128/MCB.22.12.4167-4180.2002 PMC133845

[B55] Horn D. McCulloch R. (2010). Molecular mechanisms underlying the control of antigenic variation in African trypanosomes. *Curr. Opin. Microbiol.* 13 700–705 20884281 10.1016/j.mib.2010.08.009 PMC3117991

[B56] Horn D Cross G. A. M. (1997a). Position-dependent and promoter-specific regulation of gene expression in *Trypanosoma brucei*. *EMBO J.* 16 7422–7431 9405371 10.1093/emboj/16.24.7422 PMC1170342

[B57] Horn D Cross G. A. M. (1997b). Analysis of *Trypanosoma brucei* vsg expression site switching *in vitro*. *Mol. Biochem. Parasitol.* 84 189–201 9084039 10.1016/s0166-6851(96)02794-6

[B58] Horn D. Spence C. Ingram A. K. (2000). Telomere maintenance and length regulation in *Trypanosoma brucei*. *EMBO J.* 19 2332–2339 10811624 10.1093/emboj/19.10.2332 PMC384376

[B59] Houghtaling B. R. Cuttonaro L. Chang W. Smith S. (2004). A dynamic molecular link between the telomere length regulator TRF1 and the chromosome end protector TRF2. *Curr. Biol.* 14 1621–1631 15380063 10.1016/j.cub.2004.08.052

[B60] Hovel-Miner G. A. Boothroyd C. E. Mugnier M. Dreesen O. Cross G. A. Papavasiliou F. N. (2012). Telomere length affects the frequency and mechanism of antigenic variation in *Trypanosoma brucei*. *PLoS Pathog.* 8 e1002900 10.1371/journal.ppat.1002900 PMC3431348 22952449

[B61] Hughes K. Wand M. Foulston L. Young R. Harley K. Terry S. (2007). A novel ISWI is involved in VSG expression site downregulation in African trypanosomes. *EMBO J.* 26 2400–2410 17431399 10.1038/sj.emboj.7601678 PMC1864976

[B62] Imai S. Armstrong C. M. Kaeberlein M. Guarente L. (2000). Transcriptional silencing and longevity protein Sir2 is an NAD- dependent histone deacetylase. *Nature* 403 795–800 10693811 10.1038/35001622

[B63] Iraqui I. Garcia-Sanchez S. Aubert S. Dromer F. Ghigo J. M. d’Enfert C. (2005). The Yak1p kinase controls expression of adhesins and biofilm formation in *Candida glabrata* in a Sir4p-dependent pathway. *Mol. Microbiol.* 55 1259–1271 15686569 10.1111/j.1365-2958.2004.04475.x

[B64] Janzen C. J. Hake S. B. Lowell J. E Cross G. A. M. (2006). Selective di- or trimethylation of histone H3 lysine 76 by two DOT1 homologs is important for cell cycle regulation in *Trypanosoma brucei*. *Mol. Cell* 23 497–507 16916638 10.1016/j.molcel.2006.06.027

[B65] Kapteyn J. C. Van Den Ende H. Klis F. M. (1999). The contribution of cell wall proteins to the organization of the yeast cell wall. *Biochim. Biophys. Acta* 1426 373–383 9878836 10.1016/s0304-4165(98)00137-8

[B66] Kaur R. Domergue R. Zupancic M. L. Cormack B. P. (2005). A yeast by any other name: *Candida glabrata* and its interaction with the host. *Curr. Opin. Microbiol.* 8 378–384 15996895 10.1016/j.mib.2005.06.012

[B67] Keely S. P. Renauld H. Wakefield A. E. Cushion M. T. Smulian A. G. Fosker N. (2005). Gene arrays at *Pneumocystis carinii* telomeres. *Genetics* 170 1589–1600 15965256 10.1534/genetics.105.040733 PMC1449779

[B68] Keely S. P. Stringer J. R. (2009). Complexity of the MSG gene family of *Pneumocystis carinii*. *BMC Genomics* 10 367 10.1186/1471-2164-10-367 PMC2743713 19664205

[B69] Kim H. S Cross G. A. M. (2010). TOPO3α influences antigenic variation by monitoring expression-site-associated VSG switching in *Trypanosoma brucei*. *PLoS Pathog.* 6 e1000992 10.1371/journal.ppat.1000992 PMC2900300 20628569

[B70] Kim H. S Cross G. A. M. (2011). Identification of *Trypanosoma brucei* RMI1/BLAP75 homologue and its roles in antigenic variation. *PLoS ONE* 6 e25313 10.1371/journal.pone.0025313 PMC3182221 21980422

[B71] Kim S. H. Kaminker P. Campisi J. (1999). TIN2, a new regulator of telomere length in human cells. *Nat. Genet.* 23 405–412 10581025 10.1038/70508 PMC4940194

[B72] Koering C. E. Pollice A. Zibella M. P. Bauwens S. Puisieux A. Brunori M. (2002). Human telomeric position effect is determined by chromosomal context and telomeric chromatin integrity. *EMBO Rep.* 3 1055–1061 12393752 10.1093/embo-reports/kvf215 PMC1307600

[B73] Kraemer S. M. Smith J. D. (2003). Evidence for the importance of genetic structuring to the structural and functional specialization of the *Plasmodium falciparum* var gene family. *Mol. Microbiol.* 50 1527–1538 14651636 10.1046/j.1365-2958.2003.03814.x

[B74] Labandeira-Rey M. Skare J. T. (2001). Decreased infectivity in *Borrelia burgdorferi* strain B31 is associated with loss of linear plasmid 25 or 28-1. *Infect. Immun.* 69 446–455 11119536 10.1128/IAI.69.1.446-455.2001 PMC97902

[B75] Landry J. Sutton A. Tafrov S. T. Heller R. C. Stebbins J. Pillus L. (2000). The silencing protein SIR2 and its homologs are NAD-dependent protein deacetylases. *Proc. Natl. Acad. Sci. U.S.A.* 97 5807–5811 10811920 10.1073/pnas.110148297 PMC18515

[B76] Lavstsen T. Salanti A. Jensen A. T. Arnot D. E. Theander T. G. (2003). Sub-grouping of *Plasmodium falciparum* 3D7 var genes based on sequence analysis of coding and non-coding regions. *Malar. J.* 2 27 10.1186/1475-2875-2-27 PMC222925 14565852

[B77] Lenardo M. J. Rice-Ficht A. C. Kelly G. Esser K. M. Donelson J. E. (1984). Characterization of the genes specifying two metacyclic variable antigen types in *Trypanosoma brucei rhodesiense*. *Proc. Natl. Acad. Sci. U.S.A.* 81 6642–6646 6593722 10.1073/pnas.81.21.6642 PMC391986

[B78] Lewis K. A. Wuttke D. S. (2012). Telomerase and telomere-associated proteins: structural insights into mechanism and evolution. *Structure* 20 28–39 22244753 10.1016/j.str.2011.10.017 PMC4180718

[B79] Li B. Espinal A Cross G. A. M. (2005). Trypanosome telomeres are protected by a homologue of mammalian TRF2. *Mol. Cell. Biol.* 25 5011–5021 15923618 10.1128/MCB.25.12.5011-5021.2005 PMC1140576

[B80] Li B. Oestreich S de Lange T. (2000). Identification of human Rap1: implications for telomere evolution. *Cell* 101 471–483 10850490 10.1016/s0092-8674(00)80858-2

[B81] Li B. Lustig A. J. (1996). A novel mechanism for telomere size control in *Saccharomyces cerevisiae*. *Genes Dev.* 10 1310–1326 8647430 10.1101/gad.10.11.1310

[B82] Liu D. Safari A. O’Connor M. S. Chan D. W. Laegeler A. Qin J. (2004). PTOP interacts with POT1 and regulates its localization to telomeres. *Nat. Cell Biol.* 6 673–680 15181449 10.1038/ncb1142

[B83] Longtine M. S. Wilson N. M. Petracek M. E. Berman J. (1989). A yeast telomere binding activity binds to two related telomere sequence motifs and is indistinguishable from RAP1. *Curr. Genet.* 16 225–239 2697465 10.1007/BF00422108

[B84] Louis E. J. (1995). The chromosome ends of *Saccharomyces cerevisiae*. *Yeast* 11 1553–1573 8720065 10.1002/yea.320111604

[B85] Luo K. Vega-Palas M. A. Grunstein M. (2002). Rap1-Sir4 binding independent of other Sir, yKu, or histone interactions initiates the assembly of telomeric heterochromatin in yeast. *Genes Dev.* 16 1528–1539 12080091 10.1101/gad.988802 PMC186350

[B86] Mancio-Silva L. Rojas-Meza A. P. Vargas M. Scherf A. Hernandez-Rivas R. (2008). Differential association of Orc1 and Sir2 proteins to telomeric domains in *Plasmodium falciparum*. *J. Cell Sci.* 121 2046–2053 18525026 10.1242/jcs.026427

[B87] Marcello L. Barry J. D. (2007). Analysis of the VSG gene silent archive in *Trypanosoma brucei* reveals that mosaic gene expression is prominent in antigenic variation and is favored by archive substructure. *Genome Res.* 17 1344–1352 17652423 10.1101/gr.6421207 PMC1950903

[B88] Martin S. G. Laroche T. Suka N. Grunstein M. Gasser S. M. (1999). Relocalization of telomeric Ku and SIR proteins in response to DNA strand breaks in yeast. *Cell* 97 621–633 10367891 10.1016/s0092-8674(00)80773-4

[B89] Martino F. Kueng S. Robinson P. Tsai-Pflugfelder M. van Leeuwen F. Ziegler M. (2009). Reconstitution of yeast silent chromatin: multiple contact sites and O-AADPR binding load SIR complexes onto nucleosomes in vitro. *Mol. Cell.* 33 323–334 19217406 10.1016/j.molcel.2009.01.009

[B90] Matthews K. R. (2005). The developmental cell biology of *Trypanosoma brucei*. *J. Cell Sci.* 118 283–290 15654017 10.1242/jcs.01649 PMC2686837

[B91] McCulloch R. Barry J. D. (1999). A role for RAD51 and homologous recombination in *Trypanosoma brucei* antigenic variation. *Genes Dev.* 13 2875–2888 10557214 10.1101/gad.13.21.2875 PMC317127

[B92] McEachern M. J. Haber J. E. (2006). “Telomerase-independent telomere maintenance in yeast,” in *Telomeres*, eds T. de lange, V. Lundblad, and E. H. Blackburn (Cold Spring Harbor, NY: Cold Spring Harbor Laboratory Press)

[B93] Mehlert A. Zitzmann N. Richardson J. M. Treumann A Ferguson M. A. J. (1998). The glycosylation of the variant surface glycoproteins and procyclic acidic repetitive proteins of *Trypanosoma brucei*. *Mol. Biochem. Parasitol.* 91 145–152 9574932 10.1016/s0166-6851(97)00187-4

[B94] Melville S. E. Leech V. Navarro M Cross G. A. M. (2000). The molecular karyotype of the megabase chromosomes of *Trypanosoma brucei* stock 427. *Mol. Biochem. Parasitol.* 111 261–273 11163435 10.1016/s0166-6851(00)00316-9

[B95] Merrick C. J. Duraisingh M. T. (2007). *Plasmodium falciparum* Sir2: an unusual sirtuin with dual histone deacetylase and ADP-ribosyltransferase activity. *Eukaryot. Cell* 6 2081–2091 17827348 10.1128/EC.00114-07 PMC2168398

[B96] Miyake Y. Nakamura M. Nabetani A. Shimamura S. Tamura M. Yoneha-ra S. (2009). RPA-like mamma-lian Ctc1-Stn1-Ten1 complex binds to single-stranded DNA and protects telomeres independently of the Pot1 pathway. *Mol. Cell.* 36 193–206 19854130 10.1016/j.molcel.2009.08.009

[B97] Miyoshi T. Kanoh J. Saito M. Ishikawa F. (2008). Fission yeast Pot1-Tpp1 protects telomeres and regulates telomere length. *Science* 320 1341–1344 18535244 10.1126/science.1154819

[B98] Moazed D. Kistler A. Axelrod A. Rine J. Johnson A. D. (1997). Silent information regulator protein complexes in *Saccharomyces cerevisiae* – a sir2/sir4 complex and evidence for a regulatory domain in sir4 that inhibits its interaction with sir3. *Proc. Natl. Acad. Sci. U.S.A.* 94 2186–2191 9122169 10.1073/pnas.94.6.2186 PMC20062

[B99] Moretti P. Freeman K. Coodly L. Shore D. (1994). Evidence that a complex of SIR proteins interacts with the silencer and telomere-binding protein RAP1. *Genes Dev.* 8 2257–2269 7958893 10.1101/gad.8.19.2257

[B100] Moretti P. Shore D. (2001). Multiple interactions in sir protein recruitment by Rap1p at silencers and telomeres in yeast. *Mol. Cell. Biol.* 21 8082–8094 11689698 10.1128/MCB.21.23.8082-8094.2001 PMC99974

[B101] Myler P. J. Aline R. F. Jr. Scholler J. K. Stuart K. D. (1988). Changes in telomere length associated with antigenic variation in *Trypanosoma brucei*. *Mol. Biochem. Parasitol.* 29 243–250 2842675 10.1016/0166-6851(88)90079-5

[B102] Nabetani A. Ishikawa F. (2011). Alternative lengthening of telomeres pathway: recombination-mediated telomere maintenance mechanism in human cells. *J. Biochem.* 149 5–14 20937668 10.1093/jb/mvq119

[B103] Navarro M. Gull K. (2001). A pol I transcriptional body associated with VSG mono-allelic expression in *Trypanosoma brucei*. *Nature* 414 759–763 11742402 10.1038/414759a

[B104] Norris S. J. (2006). Antigenic variation with a twist – the Borrelia story. *Mol. Microbiol.* 60 1319–1322 16796669 10.1111/j.1365-2958.2006.05204.x

[B105] Park M. J. Jang Y. K. Choi E. S. Kim H. S. Park S. D. (2002). Fission yeast Rap1 homolog is a telomere-specific silencing factor and interacts with Taz1p. *Mol. Cells* 13 327–333 12018857

[B106] Pays E. Laurent M. Delinte K. Van Meirvenne N. Steinert M. (1983a). Differential size variations between transcriptionally active and inactive telomeres of *Trypanosoma brucei*. *Nucleic Acids Res.* 11 8137–8147 6324074 10.1093/nar/11.23.8137 PMC326571

[B107] Pays E. van Assel S. Laurent M. Dero B. Michiels F. Kronenberger P. (1983b). At least two transposed sequences are associated in the expression site of a surface antigen gene in different trypanosome clones. *Cell* 34 359–369 6311429 10.1016/0092-8674(83)90370-7

[B108] Pedram M. Sprung C. N. Gao Q. Lo A. W. Reynolds G. E. Murnane J. P. (2006). Telomere position effect and silencing of transgenes near telomeres in the mouse. *Mol. Cell. Biol.* 26 1865–1878 16479005 10.1128/MCB.26.5.1865-1878.2006 PMC1430234

[B109] Perez-Toledo K. Rojas-Meza A. P. Mancio-Silva L. Hernandez-Cuevas N. A. Delgadillo D. M. Vargas M. (2009). *Plasmodium falciparum* heterochromatin protein 1 binds to tri-methylated histone 3 lysine 9 and is linked to mutually exclusive expression of var genes. *Nucleic Acids Res.* 37 2596–2606 19270070 10.1093/nar/gkp115 PMC2677873

[B110] Pham V. P. Qi C. C. Gottesdiener K. M. (1996). A detailed mutational analysis of the VSG gene expression site promoter. *Mol. Biochem. Parasitol.* 75 241–254 8992322 10.1016/0166-6851(95)02513-8

[B111] Pologe L. G. Ravetch J. V. (1988). Large deletions result from breakage and healing of *P. falciparum *chromosomes. *Cell* 55 869–874 3056622 10.1016/0092-8674(88)90142-0

[B112] Proudfoot C. McCulloch R. (2005). Distinct roles for two RAD51-related genes in *Trypanosoma brucei* antigenic variation. *Nucleic Acids Res.* 33 6906–6919 16326865 10.1093/nar/gki996 PMC1301600

[B113] Purser J. E. Norris S. J. (2000). Correlation between plasmid content and infectivity in *Borrelia burgdorferi*. *Proc. Natl. Acad. Sci. U.S.A.* 97 13865–13870 11106398 10.1073/pnas.97.25.13865 PMC17667

[B114] Riha K. Heacock M. L. Shippen D. E. (2006). The role of the nonhomologous end-joining DNA double-strand break repair pathway in telomere biology. *Annu. Rev. Genet.* 40 237–277 16822175 10.1146/annurev.genet.39.110304.095755

[B115] Roberts D. J. Craig A. G. Berendt A. R. Pinches R. Nash G. Marsh K. (1992). Rapid switching to multiple antigenic and adhesive phenotypes in malaria. *Nature* 357 689–692 1614515 10.1038/357689a0 PMC3731710

[B116] Robinson N. P. Burman N. Melville S. E. Barry J. D. (1999). Predominance of duplicative VSG gene conversion in antigenic variation in African trypanosomes. *Mol. Cell. Biol.* 19 5839–5846 10454531 10.1128/mcb.19.9.5839 PMC84433

[B117] Rudenko G. McCulloch R. Dirksmulder A. Borst P. (1996). Telomere exchange can be an important mechanism of variant surface glycoprotein gene switching in *Trypanosoma brucei*. *Mol. Biochem. Parasitol.* 80 65–75 8885223 10.1016/0166-6851(96)02669-2

[B118] Rudenko G Van der Ploeg L. H. (1989). Transcription of telomere repeats in protozoa. *EMBO J.* 8 2633–2638 2511008 10.1002/j.1460-2075.1989.tb08403.x PMC401269

[B119] Schwan T. G. Karstens R. H. Schrumpf M. E. Simpson W. J. (1991). Changes in antigenic reactivity of *Borrelia burgdorferi*, the Lyme disease spirochete, during persistent infection in mice. *Can. J. Microbiol.* 37 450–454 1913349 10.1139/m91-074

[B120] Shi H. F. Djikeng A. Mark T. Wirtz E. Tschudi C. Ullu E. (2000). Genetic interference in *Trypanosoma brucei* by heritable and inducible double-stranded RNA. *RNA* 6 1069–1076 10917601 10.1017/s1355838200000297 PMC1369981

[B121] Smith J. D. Chitnis C. E. Craig A. G. Roberts D. J. Hudson-Taylor D. E. Peterson D. S. (1995). Switches in expression of *Plasmodium falciparum* var genes correlate with changes in antigenic and cytoadherent phenotypes of infected erythrocytes. *Cell* 82 101–110 7606775 10.1016/0092-8674(95)90056-x PMC3730239

[B122] Stanne T. M. Kushwaha M. Wand M. Taylor J. E. Rudenko G. (2011). TbISWI regulates multiple polymerase I (Pol I)-transcribed loci and is present at Pol II transcription boundaries in *Trypanosoma brucei*. *Eukaryot. Cell* 10 964–976 21571922 10.1128/EC.05048-11 PMC3147422

[B123] Stanne T. M. Rudenko G. (2010). Active VSG expression sites in *Trypanosoma brucei* are depleted of nucleosomes. *Eukaryot. Cell* 9 136–147 19915073 10.1128/EC.00281-09 PMC2805301

[B124] Steere A. C. Coburn J. Glickstein L. (2004). The emergence of Lyme disease. *J. Clin. Invest.* 113 1093–1101 15085185 10.1172/JCI21681 PMC385417

[B125] Stewart J. A. Chaiken M. F. Wang F. Price C. M. (2011). Maintaining the end: roles of telomere proteins in end-protection, telomere replication and length regulation. *Mutat. Res.* 730 12–19 21945241 10.1016/j.mrfmmm.2011.08.011 PMC3256267

[B126] Strahl-Bolsinger S. Hecht A. Luo K. Grunstein M. (1997). SIR2 and SIR4 interactions differ in core and extended telomeric heterochromatin in yeast. *Genes Dev.* 11 83–93 9000052 10.1101/gad.11.1.83

[B127] Stringer J. R. (2005). “Surface antigens,” in *Pneumocystis Pneumonia* eds Walzer P. D. Cushion M. T. (New York, NY: Marcel Dekker).

[B128] Su X. Heatwole V. M. Wertheimer S. P. Guinet F. Herrfeldt J. A. Peterson D. S. (1995). The large diverse gene family var encodes proteins involved in cytoadherence and antigenic variation of *Plasmodium falciparum*-infected erythrocytes. *Cell* 82 89–100 7606788 10.1016/0092-8674(95)90055-1

[B129] Sun D. Thompson B. Cathers B. E. Salazar M. Kerwin S. M. Trent J. O. (1997). Inhibition of human telomerase by a G-quadruplex-interactive compound. *J. Med. Chem.* 40 2113–2116 9216827 10.1021/jm970199z

[B130] Sunkin S. M. Linke M. J. McCormack F. X. Walzer P. D. Stringer J. R. (1998). Identification of a putative precursor to the major surface glycoprotein of *Pneumocystis carinii*. *Infect. Immun.* 66 741–746 9453635 10.1128/iai.66.2.741-746.1998 PMC113502

[B131] Sunkin S. M. Stringer J. R. (1997). Residence at the expression site is necessary and sufficient for the transcription of surface antigen genes of *Pneumocystis carinii*. *Mol. Microbiol.* 25 147–160 11902717 10.1046/j.1365-2958.1997.4461806.x

[B132] Tanny J. C. Dowd G. J. Huang J. Hilz H. Moazed D. (1999). An enzymatic activity in the yeast Sir2 protein that is essential for gene silencing. *Cell* 99 735–745 10619427 10.1016/s0092-8674(00)81671-2

[B133] Tonkin C. J. Carret C. K. Duraisingh M. T. Voss T. S. Ralph S. A. Hommel M. (2009). Sir2 paralogues cooperate to regulate virulence genes and antigenic variation in *Plasmodium falciparum*. *PLoS Biol.* 7 e84 10.1371/journal.pbio.1000084 PMC2672602 19402747

[B134] van der Ploeg L. H. T. Liu A. Y. C. Borst P. (1984). Structure of the growing telomeres of trypanosomes. *Cell* 36 459–468 6319026 10.1016/0092-8674(84)90239-3

[B135] Vanhamme L. Poelvoorde P. Pays A. Tebabi P. Van Xong H. Pays E. (2000). Differential RNA elongation controls the variant surface glycoprotein gene expression sites of *Trypanosoma brucei*. *Mol. Microbiol.* 36 328–340 10792720 10.1046/j.1365-2958.2000.01844.x

[B136] Voss T. S. Thompson J. K. Waterkeyn J. Felger I. Weiss N. Cowman A. F. (2000). Genomic distribution and functional characterisation of two distinct and conserved *Plasmodium falciparum* var gene 5′ flanking sequences. *Mol. Biochem. Parasitol.* 107 103–115 10717306 10.1016/s0166-6851(00)00176-6

[B137] Wada M. Sunkin S. M. Stringer J. R. Nakamura Y. (1995). Antigenic variation by positional control of major surface glycoprotein gene expression in *Pneumocystis carinii*. *J. Infect. Dis.* 171 1563–1568 7769293 10.1093/infdis/171.6.1563

[B138] Wada M. Nakamura Y. (1996). Unique telomeric expression site of major-surface-glycoprotein genes of *Pneumocystis carinii*. *DNA Res.* 3 55–64 8804856 10.1093/dnares/3.2.55

[B139] Wan M. Qin J. Songyang Z. Liu D. (2009). OB fold-containing protein 1 (OBFC1), a human homolog of yeast Stn1, associates with TPP1 and is implicated in telomere length regulation. *J. Biol. Chem.* 284 26725–26731 19648609 10.1074/jbc.M109.021105 PMC2785360

[B140] Wang Q. P. Kawahara T. Horn D. (2010). Histone deacetylases play distinct roles in telomeric VSG expression site silencing in African trypanosomes. *Mol. Microbiol.* 77 1237–1245 20624217 10.1111/j.1365-2958.2010.07284.x PMC2941730

[B141] Yang X. Figueiredo L. M. Espinal A. Okubo E. Li B. (2009). RAP1 is essential for silencing telomeric variant surface glycoprotein genes in *Trypanosoma brucei*. *Cell* 137 99–109 19345190 10.1016/j.cell.2009.01.037 PMC2673096

[B142] Ye J. Z. Hockemeyer D. Krutchinsky A. N. Loayza D. Hooper S. M. Chait B. T. (2004). POT1-interacting protein PIP1: a telomere length regulator that recruits POT1 to the TIN2/TRF1 complex. *Genes Dev.* 18 1649–1654 15231715 10.1101/gad.1215404 PMC478187

[B143] Zhang J. R. Hardham J. M. Barbour A. G. Norris S. J. (1997). Antigenic variation in Lyme disease borreliae by promiscuous recombination of VMP-like sequence cassettes. *Cell* 89 275–285 9108482 10.1016/s0092-8674(00)80206-8

[B144] Zhang J. R. Norris S. J. (1998a). Genetic variation of the *Borrelia burgdorferi* gene vlsE involves cassette-specific, segmental gene conversion. *Infect. Immun.* 66 3698–3704 9673251 10.1128/iai.66.8.3698-3704.1998 PMC108404

[B145] Zhang J. R. Norris S. J. (1998b). Kinetics and *in vivo* induction of genetic variation of vlsE in *Borrelia burgdorferi*. *Infect. Immun.* 66 3689–3697 9673250 10.1128/iai.66.8.3689-3697.1998 PMC108403

[B146] Zomerdijk J. C. B. M. Kieft R. Duyndam M. Shiels P. G. Borst P. (1991). Antigenic variation in *Trypanosoma brucei*: a telomeric expression site for variant-specific surface glycoprotein genes with novel features. *Nucleic Acids Res.* 19 1359–1368 1709274 10.1093/nar/19.7.1359 PMC333887

[B147] Zomerdijk J. C. B. M. Ouellete M. ten Asbroek A. L. M. Kieft R. Bommer A. M. M. Clayton C. E. (1990). The promoter for a variant surface glycoprotein gene expression site in *Trypanosoma brucei*. *EMBO J.* 9 2791–2801 1697265 10.1002/j.1460-2075.1990.tb07467.x PMC551989

